# Graph Theoretical Approaches in Brain Networks

**DOI:** 10.1155/2012/590483

**Published:** 2012-12-17

**Authors:** Fabrizio De Vico Fallani, Danielle Bassett, Tianzi Jiang

**Affiliations:** ^1^Research Centre of the Brain and Spinal Cord Institute, INSERM/UPMC/CNRS, 75005 Paris, France; ^2^Department of Physiology and Pharmacology, Sapienza University of Rome, 00185 Rome, Italy; ^3^Department of Physics and Sage Center for the Study of the Mind, University of California, Santa Barbara, CA 93106, USA; ^4^National Laboratory of Pattern Recognition, Institute of Automation, Chinese Academy of Sciences, Beijing 100190, China

In the last decade network theory has proved an effective tool for modeling and describing the complex topology emerging either from anatomical or functional brain connectivity patterns. From a graph theoretical perspective, the brain can be conceived as a networked system composed of nodes coincident with different brain sites and links which in the current view can either represent anatomical tracts between brain regions or measures of statistical dependencies between their electrical activity. One of the most intriguing, and now well known, examples of the application of network theory to neuroimaging data unveiled that the way brain regions are connected is typically neither regular nor random. Instead brain networks, like other real networked systems, tend to exhibit a complex structure theoretically consistent with the capability of processing information within regional clusters and avoiding excessive connections between clusters. While the simplest instantiation of this configuration showed similar characteristics to mathematically defined “small-world” networks, it has become clear that additional topological structures including hierarchical modularity can nuance our expectations of the brain's underlying architecture and dynamics. An important goal in these research endeavors is to identify how brain network organization can inform our understanding of the brain's intuitive need to balance the two competing principles of integration and segregation and how alterations in brain structure and dynamics can lead to alterations in human behavior and cognitive function. The present special issue collects a series of selected contributions related to the methodological and practical applications of network theory to anatomical and functional brain connectivity patterns. 

The contribution entitled “*Voxel scale complex networks of functional connectivity in the rat brain: neurochemical state dependence of global and local topological properties*” addresses the dependence of the functional connectivity estimated from the fMRI signals of the rat brain in response to alterations of the neurotransmitter system as induced by the administration of specific pharmaceutical drugs such as d-amphetamine, fluoxetine, and nicotine.

The contribution entitled “*A signal-processing-based approach to time-varying graph analysis for dynamic brain network identification*” proposes a dynamic network summarization approach to describe the time-varying evolution of connectivity patterns in functional brain activity. The proposed method is evaluated on event-related potential (ERP) data, which demonstrates the dynamic nature of functional connectivity.

The contribution entitled “*how the statistical validation of functional connectivity patterns can prevent erroneous definition of small-world properties of a brain connectivity network*” addresses important methodological choices that are often made in the construction of functional brain network from EEG data, including the choice of statistical thresholds to determine the presence or absence of network links and the role of spatial correlations in determining graph properties.

The contribution entitled “*weighted phase lag index and graph analysis: preliminary investigation of functional connectivity during resting state in children*” presents original results concerning the application of small-world parameters and betweenness centrality measures to characterize the topological structure of the functional network in the children's brain from noninvasive MEG recordings.

The contribution entitled “*source space analysis of event-related dynamic reorganization of brain networks*” conducts a quantitative study of the dynamic reconfiguration of connectivity for event-related experiments at source space level, which provides a global and complete view of the stages of processing associated with the regional changes in activity.

The contribution entitled “*Redundancy as a Graph-Based Index of Frequency Specific MEG Functional Connectivity*” focuses on quantifying the differential role that paths of different lengths potentially play in brain connectivity and demonstrates that a redundancy-based measure captures unique information not accessible via approaches that only examine the shortest paths through a network.

The contribution entitled “*A computationally efficient, exploratory approach to brain connectivity incorporating false discovery rate control, a priori knowledge, and group inference*” proposes a multisubject, exploratory brain connectivity modeling approach. The proposed method allows for the incorporation of prior knowledge of connectivity and the determination of the dominant brain connectivity pattern among a group of subjects.

*Fabrizio De Vico Fallani* *Danielle Bassett* *Tianzi Jiang*

